# Supplementary material for: Targeting local lymphatics to ameliorate heterotopic ossification via FGFR3-BMPR1a pathway
Source: Nat Commun. 2021 Jul 19;12:4391. doi: 10.1038/s41467-021-24643-2 (PMC8289847; doi:10.1038/s41467-021-24643-2)
Supplement: Supplementary file 1 — Supplementary Information [file 41467_2021_24643_MOESM1_ESM.pdf]

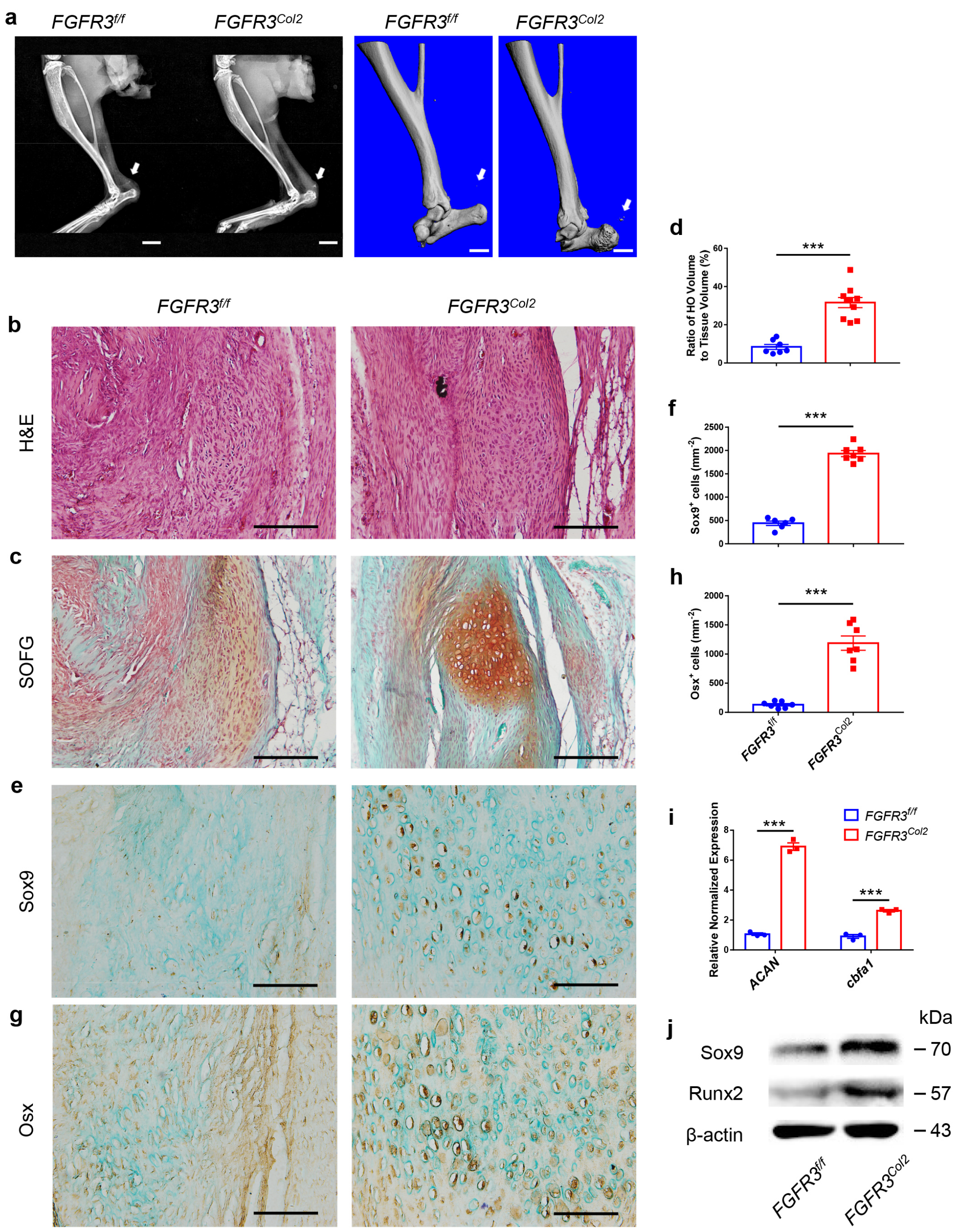

Supplementary Fig. 1 *FGFR3*^{Col2} mice have aggravated acquired HO formation at 4 weeks after Achilles tenotomy. a, Representative X-ray (left) and μCT (right) images of heterotopic bone in the Achilles tendon of *FGFR3*$^{f/f}$ and *FGFR3*^{Col2} mice at 4 weeks after tenotomy. n=6 per group. White arrow shows ectopic bone. Scale bars, 2 mm for X-ray; 1 mm for μCT. b-d, Representative H&E and SOFG images of heterotopic bone in *FGFR3*$^{f/f}$ (n=7) and *FGFR3*^{Col2} mice (n=10) at 4 weeks after tenotomy and histomorphometry analysis. Scale bars, 200 μm. e-

h, Representative immunohistochemical staining of Sox9 (e, *FGFR3*$^{f/f}$=6, *FGFR3*^{Col2}=7) and Osx (g, *FGFR3*$^{f/f}$=7, *FGFR3*^{Col2}=7) and relative quantitative analysis (f,h). Scale bars, 100 μm. i, mRNA levels of *ACAN* and *cbfa1* in the tendon of *FGFR3*$^{f/f}$ and *FGFR3*^{Col2} mice at 4 weeks post Achilles tenotomy. n=3 per group. j, Protein levels of Sox9 and Runx2 in the Achilles tendon of *FGFR3*$^{f/f}$ and *FGFR3*^{Col2} mice at 4 weeks post surgery. n=3 per group. All data represent mean ± SEM. ***P < 0.001 by unpaired two-tailed Student's t-test.

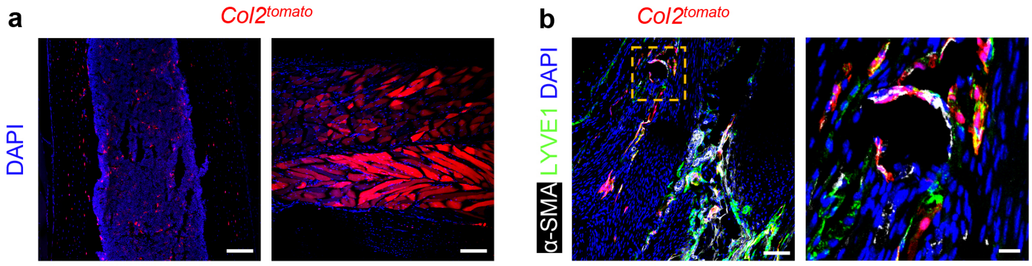

Supplementary Fig. 2 Col2^{+} lineage cells are detectable in the bone marrow, cortical bone, muscles and repaired Achilles tendon. a, Representative confocal images of tibia sections (left) and muscle sections (right) from the paws of *Col2*^{tomato} mice at 8 weeks after tamoxifen induction. n=5 per group. Blue indicates DAPI staining. Scale bars, 100 μm. b, Representative confocal images of repaired Achilles tendon immunostained with α-SMA (white), LYVE1 (green) and DAPI (blue) in *Col2*^{tomato} mice (n=4) at 8 weeks after surgery. Dashed line boxes indicate the site of tdTomato-labeled cells stained by α-SMA (higher magnification, right). Scale bars, 100 μm (left); 20 μm (right).

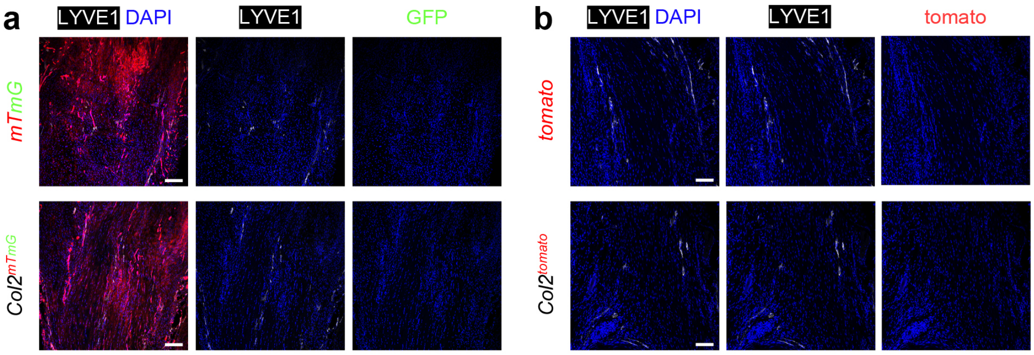

Supplementary Fig. 3 Tamoxifen-independent activation of reporter genes in *mTmG* and *tomato* lines is not observed in the repaired Achilles tendon. a,b, Representative confocal images (left) with split channels (middle and right) of repaired Achilles tendon immunostained with LYVE1 (white) and DAPI (blue) in *mTmG* and $Col2^{mTmG}$ (a) as well as *tomato* and $Col2^{tomato}$ mice (b) without tamoxifen induction at 4 weeks post tenotomy. n=3-4 per group. Scale bars, 100 µm.

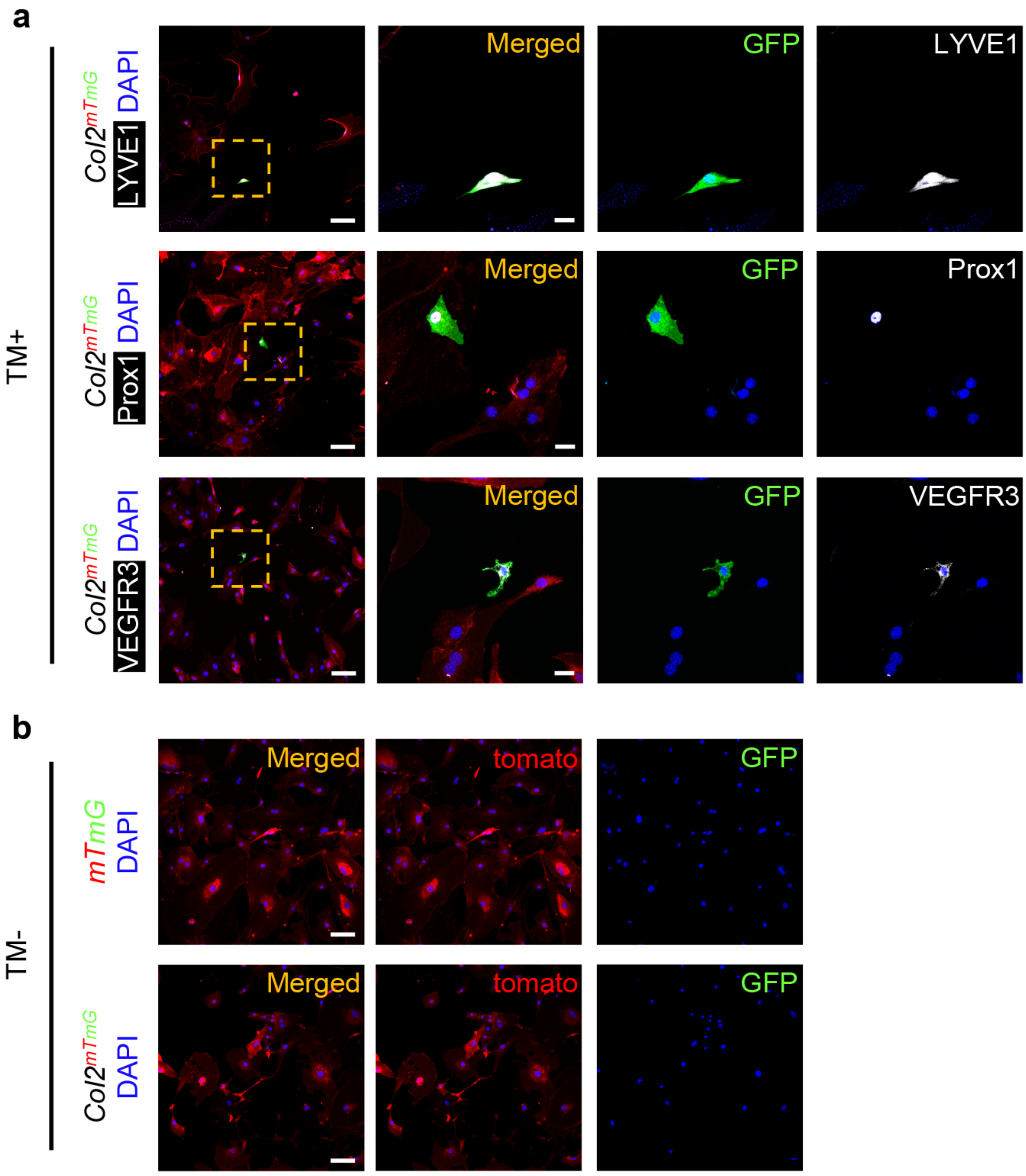

Supplementary Fig. 4 Col2-derived cells in the repaired tendon of *Col2^{mTmG}* mice are labeled by LEC markers *in vitro*. a, Representative confocal images of primary cells isolated from repaired Achilles tendons of *Col2^{mTmG}* mice at 4 weeks after tenotomy immunostained with LYVE1, Prox1 or VEGFR3 (white) and DAPI (blue). n=4 per group. Dashed line boxes indicate GFP-labeled primary cells (higher magnification with split channels, right). Scale bars, 100 µm (left); 20 µm (right). b, Representative confocal images (left) with split channels (middle and right) of primary cells isolated from repaired Achilles tendons of *mTmG* and *Col2^{mTmG}* mice

without tamoxifen induction at 4 weeks post tenotomy. n=3 per group. Scale bars, 100 µm.

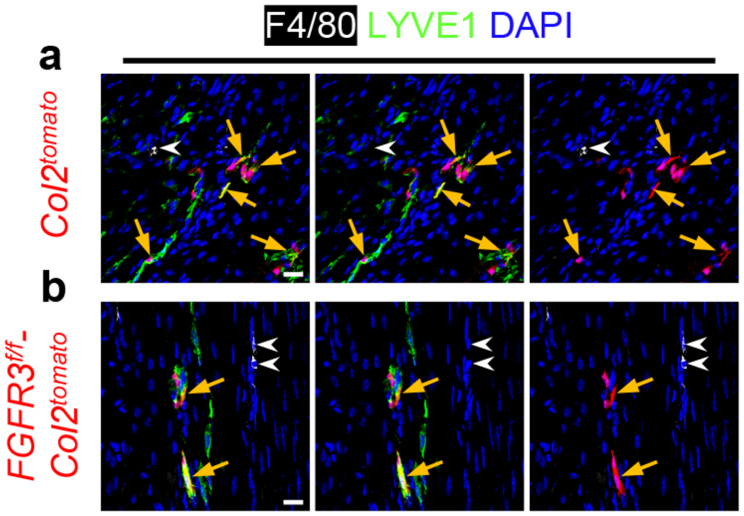

Supplementary Fig. 5 Col2^{+} lineage cells are labeled by LYVE1 instead of F4/80 in the repaired Achilles tendon. a,b, Representative confocal images (left) with split channels (middle and right) of repaired Achilles tendon immunostained with F4/80 (white), LYVE1 (green) and DAPI (blue) in *Col2^{tomato}* (a) and *FGFR3$^{f/f}$-Col2^{tomato}* mice (b) at 8 weeks after surgery. n=4 per group. Scale bars, 20 µm. Yellow arrows indicate LYVE1^{+}F4/80^{-} LECs. White arrowheads indicate LYVE1^{-}F4/80^{+} macrophages.

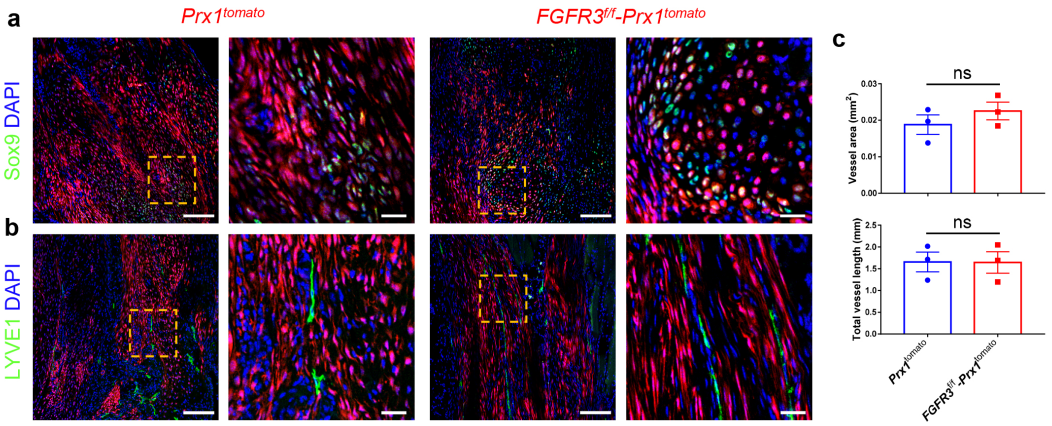

Supplementary Fig. 6 Prx1^{+} mesenchymal progenitors are directly involved in HO formation

instead of lymphatic formation in the Achilles tendon after surgery. a, Representative confocal images of ectopic bone sections immunostained with Sox9 (green) and DAPI (blue) in *Prx1^{tomato}* and *FGFR3$^{f/f}$-Prx1^{tomato}* mice at 8 weeks after Achilles tenotomy. n=3 per group. Dashed line boxes indicate Sox9^{+} tdTomato-labeled cells (higher magnification, right). Scale bars, 100 μm (left); 20 μm (right). b,c, Representative confocal images of repaired Achilles tendon immunostained with LYVE1 (green) and DAPI (blue) and relative quantitative analysis of lymphatic vessels (c) in *Prx1^{tomato}* and *FGFR3$^{f/f}$-Prx1^{tomato}* mice at 8 weeks after surgery. n=3 per group. Dashed line boxes indicate tdTomato-labeled cells and LYVE1^{+} lymphatics (higher magnification, right). Scale bars, 100 μm (left); 20 μm (right). All data represent mean ± SEM. Statistical significance was analyzed by unpaired two-tailed Student's t-test.

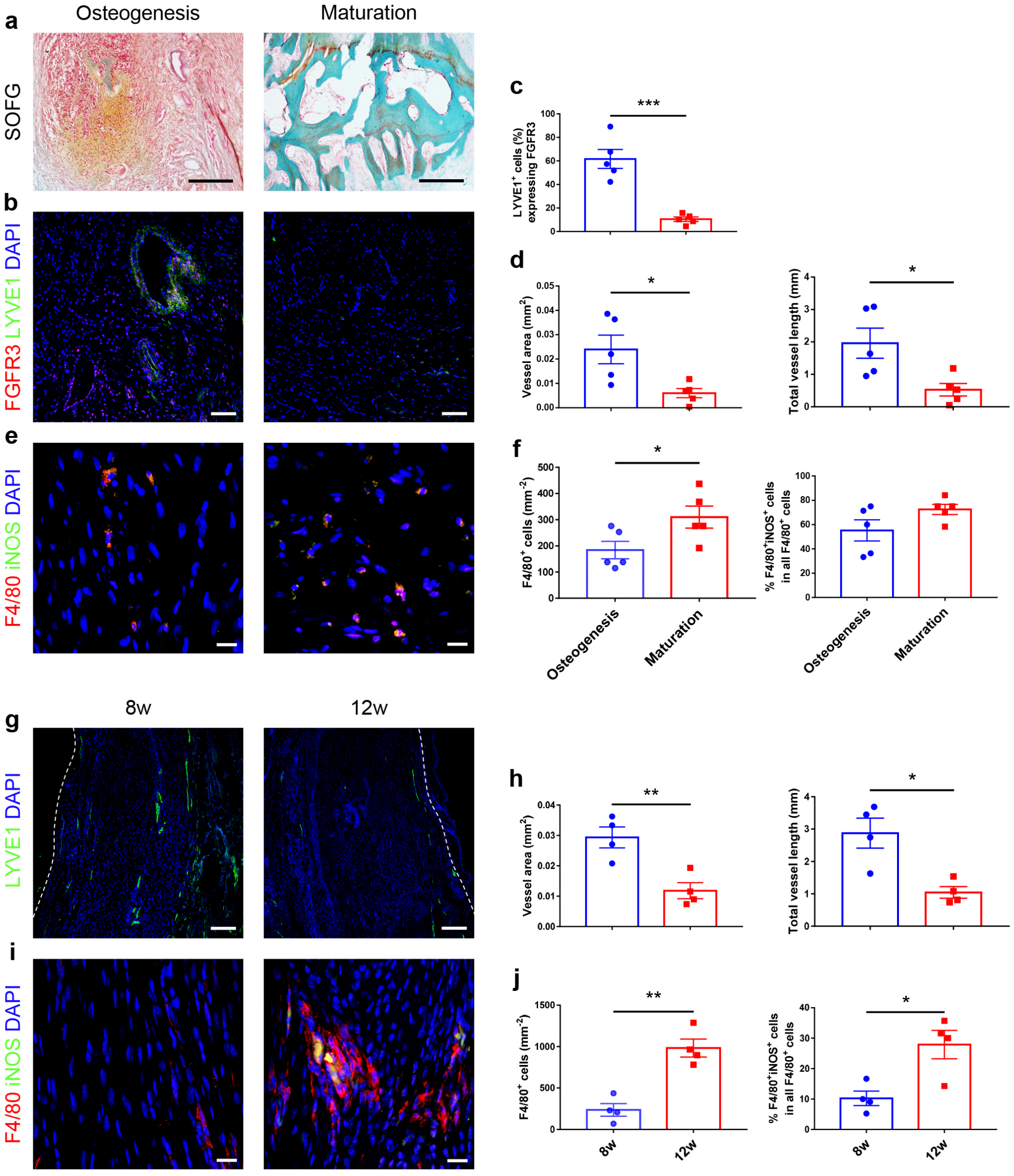

Supplementary Fig. 7 Lymphatic formation is reduced with downregulated FGFR3 expression in LECs during acquired HO progression. a, Representative SOFG images of human acquired HO in early osteogenesis stage (left, n=5) and late maturation stage (right, n=5). Scale bars, 200 μm. b-d, Representative confocal images of human HO lesions immunostained with FGFR3 (red), LYVE1 (green) and DAPI (blue) during early osteogenesis stage (left, n=5) and late maturation stage (right, n=5) and relative quantitative analysis (c,d). Scale bars, 100 μm. e,f, Representative confocal images of F4/80 (red), iNOS (green) and DAPI (blue) staining in the

sections of human HO specimens in osteogenesis stage (left, n=5) and maturation stage (right, n=5) and relative quantification (f). Scale bars, 20 μm. g,h, Representative confocal images of LYVE1 (green) and DAPI (blue) immunostained Achilles tendon sections from WT mice at 8 (left) and 12 weeks (right) after surgery and lymphatic quantitative analysis (h). White dashed lines indicate outlines of the tendon (g). n=4 per group. Scale bars, 100 μm. i,j, Representative confocal images of repaired Achilles tendon immunostained with F4/80 (red), iNOS (green) and DAPI (blue) at 8 (left) and 12 weeks (right) after tenotomy and relative quantification (j). n=4 per group. Scale bars, 20 μm. All data represent mean ± SEM. $*P < 0.05$; $**P < 0.01$; $***P < 0.001$ by unpaired two-tailed Student's t-test.

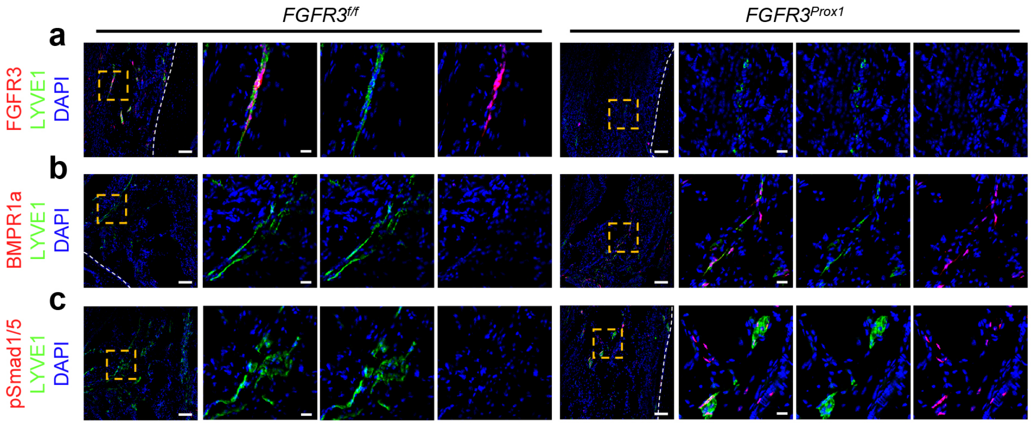

Supplementary Fig. 8 Elevated BMPR1a-pSmad1/5 signaling correlates with *FGFR3* deletion in LECs in the repaired Achilles tendon of $FGFR3^{Prox1}$ mice. a-c, Representative confocal images of repaired Achilles tendon immunostained with FGFR3 (a), BMPR1a (b) or pSmad1/5 (c) (red), LYVE1 (green) and DAPI (blue) in $FGFR3^{f/f}$ (left) and $FGFR3^{Prox1}$ mice (right) at 8 weeks after surgery. n=4 per group. Dashed line boxes indicate $LYVE1^{+}$ LECs (higher magnification with split channels, right). White dashed lines indicate outlines of the tendon. Scale bars, 100 μm (left); 20 μm (right).

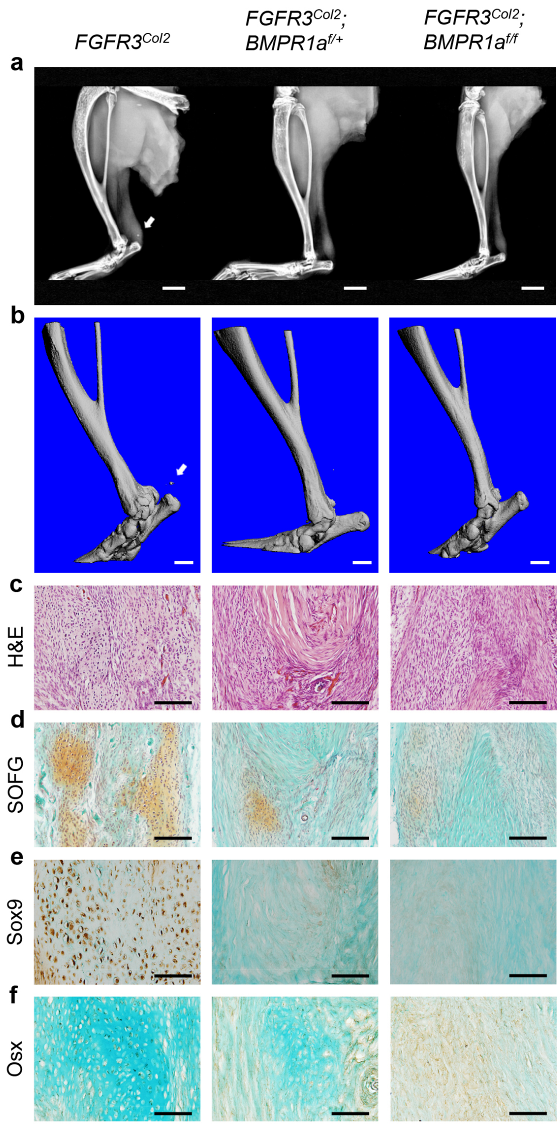

Supplementary Fig. 9 *BMPR1a* deletion alleviates HO aggravation in *FGFR3*^{Col2} mice at 4 weeks after Achilles tenotomy. a,b, Representative X-ray (a) and μCT (b) images of ectopic bone in the Achilles tendon of *FGFR3*^{Col2} (n=4), *FGFR3*^{Col2}*;BMPR1a*$^{f/+}$ (n=3) and *FGFR3*^{Col2}*;BMPR1a*$^{f/f}$ mice (n=3) at 4 weeks after surgery. Scale bars, 2 mm for X-ray; 1 mm for μCT. c,d, Representative H&E (c) and SOFG (d) staining images of Achilles tendon sections in *FGFR3*^{Col2} (n=4), *FGFR3*^{Col2}*;BMPR1a*$^{f/+}$ (n=3) and *FGFR3*^{Col2}*;BMPR1a*$^{f/f}$ mice (n=3) at 4 weeks after surgery. Scale bars, 200 μm. e,f, Representative Sox9 (e) and Osx (f) IHC images

of Achilles tendon sections. Scale bars, 100 μm.

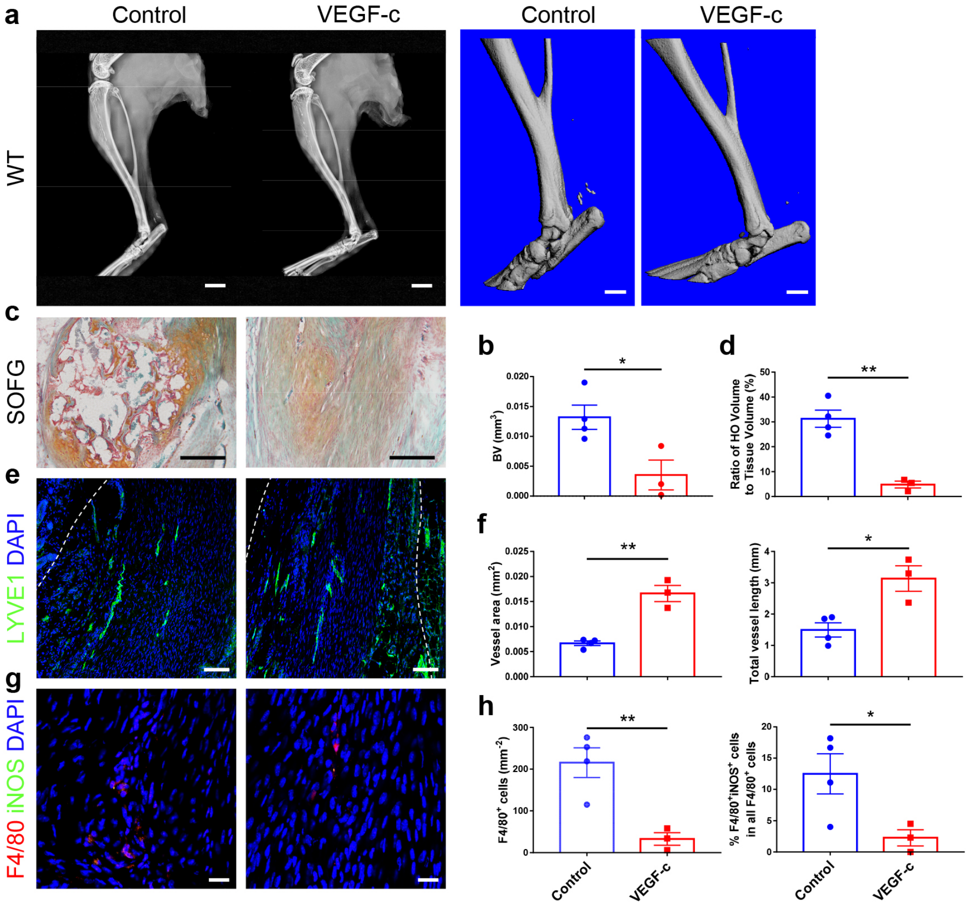

Supplementary Fig. 10 Local VEGF-c treatment inhibits HO development via promoting lymphatic formation. a,b, Representative X-ray (left) and μCT (right) images (a) of ectopic bone in the Achilles tendon of WT mice treated with VEGF-c (n=3) relative to controls (n=4) at 8 weeks after tenotomy and quantitative analysis (b). Scale bars, 2 mm for X-ray; 1 mm for μCT. c,d, Representative SOFG staining images (c) of ectopic bone in the tendon and histomorphometry analysis (d). Scale bars, 200 μm. e,f, Representative confocal images of LYVE1 (green) and DAPI (blue) immunostained Achilles tendon sections (e) and relative lymphatic quantification (f). White dashed lines indicate outlines of the tendon (e). Scale bars, 100 μm. g,h, Representative confocal images of Achilles tendon sections immunostained with

F4/80 (red), iNOS (green) and DAPI (blue) (g) in WT mice treated with VEGF-c at 8 weeks after surgery and relative quantitative analysis (h). Scale bars, 20 μm. All data represent mean ± SEM. *$P < 0.05$; **$P < 0.01$ by unpaired two-tailed Student's t-test.

Supplementary Table 1. AHO incidence in mice with *FGFR3* and *BMPR1a* deficiency.

Weeks post Achilles tenotomy	AHO incidence (%)			
	FGFR3$^{f/f}$	*FGFR3*^{Col2}	*FGFR3*^{Col2}; *BMPR1a*$^{f/+}$	*FGFR3*^{Col2}; *BMPR1a*$^{f/f}$
2	4.3 (1/23)	18.2 (4/22)	0 (0/8)	0 (0/6)
4	13.0 (3/23)	22.7 (5/22)	0 (0/8)	0 (0/6)
6	47.8 (11/23)	68.2 (15/22)	25.0 (2/8)	0 (0/6)
8	78.3 (18/23)	95.5 (21/22)	75.0 (6/8)	33.3 (2/6)

Supplementary Table 2.

Gene	Forward	Reverse
cyclophilin A	CGAGCTCTGAGCACTGGAGA	TGGCGTGTAAAGTCACCACC
ACAN	CCTGCTACTTCATCGACCCC	AGATGCTGTTGACTCGAACCT
OC	TCTGACAAAGCCTTCATGTCC	AAATAGTGATACCGTAGATGCG
cbfa1	CCTGAACTCTGCACCAAGTC	GAGGTGGCAGTGTCATCATC
LYVE1	CAGCACACTAGCCTGGTGTTA	CGCCCATGATTCTGCATGTAGA
Prox1	AGAAGGGTTGACATTGGAGTGA	TGCGTGTTGCACCACAGAATA
PDPN	ACCGTGCCAGTGTTGTTCTG	AGCACCTGTGGTTGTTATTTGT
