# Supplementary material for: Targeting local lymphatics to ameliorate heterotopic ossification via FGFR3-BMPR1a pathway
Source: Nat Commun. 2021 Jul 19;12:4391. doi: 10.1038/s41467-021-24643-2 (PMC8289847; doi:10.1038/s41467-021-24643-2)
Supplement: Supplementary file 3 — Reporting Summary [file 41467_2021_24643_MOESM3_ESM.pdf]

nature research

Corresponding author(s): Huabing Qi, Yangli Xie, Lin Chen

Last updated by author(s): Apr 15, 2021

Reporting Summary

Nature Research wishes to improve the reproducibility of the work that we publish. This form provides structure for consistency and transparency in reporting. For further information on Nature Research policies, see our Editorial Policies and the Editorial Policy Checklist.

Statistics

For all statistical analyses, confirm that the following items are present in the figure legend, table legend, main text, or Methods section.

n/a	Confirmed	
☐	☒	The exact sample size (*n*) for each experimental group/condition, given as a discrete number and unit of measurement
☐	☒	A statement on whether measurements were taken from distinct samples or whether the same sample was measured repeatedly
☐	☒	The statistical test(s) used AND whether they are one- or two-sided<br>*Only common tests should be described solely by name; describe more complex techniques in the Methods section.*
☒	☐	A description of all covariates tested
☒	☐	A description of any assumptions or corrections, such as tests of normality and adjustment for multiple comparisons
☐	☒	A full description of the statistical parameters including central tendency (e.g. means) or other basic estimates (e.g. regression coefficient) AND variation (e.g. standard deviation) or associated estimates of uncertainty (e.g. confidence intervals)
☐	☒	For null hypothesis testing, the test statistic (e.g. *F*, *t*, *r*) with confidence intervals, effect sizes, degrees of freedom and *P* value noted<br>*Give P values as exact values whenever suitable.*
☒	☐	For Bayesian analysis, information on the choice of priors and Markov chain Monte Carlo settings
☒	☐	For hierarchical and complex designs, identification of the appropriate level for tests and full reporting of outcomes
☒	☐	Estimates of effect sizes (e.g. Cohen's *d*, Pearson's *r*), indicating how they were calculated

Our web collection on statistics for biologists contains articles on many of the points above.

Software and code

Policy information about availability of computer code

Data collection	ZEISS ZEN 2.1 (black edition) Imaging Software was used for data collection with confocal laser scanning microscopy. MX-20 Cabinet X-ray system (Faxitron X-Ray, Tucson, AZ, USA) was used for X-ray imaging. VivaCT 40 µCT system (Scanco Medical, Brüttisellen, Switzerland) was used for µCT imaging.
Data analysis	GraphPad Prism 7.0 (GraphPad Software, Inc.) was used for the statistical analysis of the data and to present data in graphs. The ICG NIR images were analyzed using Evolution-Capt v18.02 software. ImageJ V1.51 was used to length and size measurements.

For manuscripts utilizing custom algorithms or software that are central to the research but not yet described in published literature, software must be made available to editors and reviewers. We strongly encourage code deposition in a community repository (e.g. GitHub). See the Nature Research guidelines for submitting code & software for further information.

Data

Policy information about availability of data

All manuscripts must include a data availability statement. This statement should provide the following information, where applicable:

- Accession codes, unique identifiers, or web links for publicly available datasets
- A list of figures that have associated raw data
- A description of any restrictions on data availability

The raw data that support the findings of this study are available from the corresponding author upon reasonable request.

Field-specific reporting

Please select the one below that is the best fit for your research. If you are not sure, read the appropriate sections before making your selection.

- [x] Life sciences
- [ ] Behavioural & social sciences
- [ ] Ecological, evolutionary & environmental sciences

For a reference copy of the document with all sections, see nature.com/documents/nr-reporting-summary-flat.pdf

Life sciences study design

All studies must disclose on these points even when the disclosure is negative.

Sample size	Sample size were chosen based on prior knowledge from previous experiments and it is generally considered that a sample size containing at least 3 biological replicates can provide adequate statistical power in biochemical analysis (ref, PMID:33658717). We have described the exact sample size for each experiment in our manuscript.
Data exclusions	No data captured was excluded from the subsequent analysis.
Replication	The exact number of replication for all experiments was described in figure legends and our attempts at replication were successful.
Randomization	Samples and animals were allocated randomly.
Blinding	The investigators were blinded to the animal genotype, group allocation and data collection/analysis by labeling different groups with letters/ numbers.

Reporting for specific materials, systems and methods

We require information from authors about some types of materials, experimental systems and methods used in many studies. Here, indicate whether each material, system or method listed is relevant to your study. If you are not sure if a list item applies to your research, read the appropriate section before selecting a response.

Materials & experimental systems

n/a	Involved in the study	
☐	☒	Antibodies
☐	☒	Eukaryotic cell lines
☒	☐	Palaeontology and archaeology
☐	☒	Animals and other organisms
☐	☒	Human research participants
☒	☐	Clinical data
☒	☐	Dual use research of concern

Methods

n/a	Involved in the study	
☒	☐	ChIP-seq
☒	☐	Flow cytometry
☒	☐	MRI-based neuroimaging

Antibodies

Antibodies used	For immunofluorescence: Sox9 (1:200; ab185230, Abcam), Runx2 (1:100; NBP1-77461, Novusbio), CD31 (1:100; FAB3628G, R&D Systems), LYVE1 (1:200; ab14917, Abcam/1:100; 14-0443-82, eBioscience),Prox1 (1:500; ab199359, Abcam), PDPN (1:200; ab11936, Abcam), VEGFR3 (1:100; AF743, R&D Systems), F4/80 (1:200; ab6640, Abcam), iNOS (1:50; ab15323, Abcam), α-SMA (1:200; A2547, Sigma-Aldrich), FGFR3 (1:100; BS90509, bioworld/1:50; sc-390423, Santa Cruz), BMPR1a (1:100; ab38560, Abcam), pSmad1/5 (1:100; 700047, Thermo); For immunohistochemistry: Sox9 (1:200; ab185230, Abcam), Osx (1:100; sc-22538, Santa Cruz), Runx2 (1:100; NBP1-77461, Novusbio), OC (1:100; sc-365797, Santa Cruz); For Western blot, Sox9 (1:2000; ab185230, Abcam), Runx2 (1:2000; NBP1-77461, Novusbio), FGFR3 (1:2000; BS90509, bioworld), BMPR1a (1:1000; ab38560, Abcam), pSmad1/5 (1:500; 700047, Thermo), Smad1/5 (1:1000; ab75273, Abcam), β-actin (1:5000; A8481, Sigma-Aldrich). Secondary antibodies: Alexa Fluor 488 donkey anti-rabbit IgG (A21206), Alexa Fluor 568 goat anti-rabbit IgG (A11011), Alexa Fluor 647 goat anti-rabbit IgG (A21245), Alexa Fluor 488 donkey anti-goat IgG (A11055), Alexa Fluor 647 rabbit anti-goat IgG (A27018), Alexa Fluor 488 goat anti-rat IgG (A11006), Alexa Fluor 568 goat anti-rat IgG (A11077), Alexa Fluor 647 goat anti-rat IgG (A21247), Alexa Fluor 488 goat anti-mouse IgG (A11001), Alexa Fluor 594 donkey anti-mouse IgG (A21203) and Alexa Fluor 647 goat anti-mouse IgG (A32728) were from Invitrogen and Alexa Fluor 647 goat anti-Syrian hamster IgG (ab180117) was from Abcam with dilution of 1:500 during immunostaining experiments.
Validation	All antibodies are from commercial sources and their validation data are available on the manufacturers' websites and described below. The appropriate dilution for all the antibodies was determined through preliminary experiments.<br>For immunofluorescence:<br>Sox9 (1:200; ab185230, Abcam), Suitable for: ICC/IF, WB, IHC-P, Flow Cyt, IP; Reacts with: Mouse, Rat, Human<br>Runx2 (1:100; NBP1-77461, Novusbio), Suitable for: WB, ICC/IF, IHC, IHC-P; Reacts with: Hu, Mu<br>CD31 (1:100; FAB3628G, R&D Systems), Suitable for: Flow Cytometry; Reacts with: Mouse/Rat

LYVE1 (1:200; ab14917, Abcam), Suitable for: ICC, IHC-P; Reacts with: Mouse
LYVE1 (1:100; 14-0443-82, eBioscience), Suitable for: IHC, IHC (F), ICC, IF, Flow; Reacts with: Mouse
Prox1 (1:500; ab199359, Abcam), Suitable for: ICC/IF, WB, IHC-P; Reacts with: Mouse, Rat, Human
PDPN (1:200; ab11936, Abcam), Suitable for: IHC-P; Reacts with: Mouse
VEGFR3 (1:100; AF743, R&D Systems), Suitable for: Western Blot, Flow Cytometry, CyTOF-ready; Reacts with: Mouse
F4/80 (1:200; ab6640, Abcam), Suitable for: Flow Cyt, ICC/IF; Reacts with: Mouse
iNOS (1:50; ab15323, Abcam), Suitable for: IHC-P, WB; Reacts with: Mouse
α-SMA (1:200; A2547, Sigma-Aldrich), Suitable for: IF, IHC (f), IHC (p), WB; Reacts with: human, frog, sheep, chicken, goat, bovine, rat, guinea pig, mouse, canine, rabbit, snake
FGFR3 (1:100; BS90509, bioworld), Suitable for: WB ICC/IF IHC FC; Reacts with: Human, Mouse, Rat
FGFR3 (1:50; sc-390423, Santa Cruz), Suitable for: WB, IP, IF, ELISA; Reacts with: mouse, rat, human
BMPR1a (1:100; ab38560, Abcam), Suitable for: IHC-P, IHC-Fr, ICC/IF, WB; Reacts with: Mouse, Rat, Human
pSmad1/5 (1:100; 700047, Thermo), Suitable for: WB, IHC, IHC (P), ICC, IF, Flow; Reacts with: Human, Fish, Mammal, Mouse
For immunohistochemistry:
Sox9 (1:200; ab185230, Abcam), Suitable for: ICC/IF, WB, IHC-P, Flow Cyt, IP; Reacts with: Mouse, Rat, Human
Osx (1:100; sc-22538, Santa Cruz), Suitable for: ICC/IF, WB; Reacts with: Mouse, Rat, Human
Runx2 (1:100; NBP1-77461, Novusbio), Suitable for: WB, ICC/IF, IHC, IHC-P; Reacts with: Hu, Mu
OC (1:100; sc-365797, Santa Cruz), Suitable for: WB, IP, IF, IHC(P), ELISA; Reacts with: mouse, rat, human;
For Western blot,
Sox9 (1:2000; ab185230, Abcam), Suitable for: ICC/IF, WB, IHC-P, Flow Cyt, IP; Reacts with: Mouse, Rat, Human
Runx2 (1:2000; NBP1-77461, Novusbio), Suitable for: WB, ICC/IF, IHC, IHC-P; Reacts with: Hu, Mu
FGFR3 (1:2000; BS90509, bioworld), Suitable for: WB ICC/IF IHC FC; Reacts with: Human, Mouse, Rat
BMPR1a (1:1000; ab38560, Abcam), Suitable for: IHC-P, IHC-Fr, ICC/IF, WB; Reacts with: Mouse, Rat, Human
pSmad1/5 (1:500; 700047, Thermo), Suitable for: WB, IHC, IHC (P), ICC, IF, Flow; Reacts with: Human, Fish, Mammal, Mouse
Smad1/5 (1:1000; ab75273, Abcam), Suitable for: Flow Cyt, WB; Reacts with: Rat, Human
β-actin (1:5000; A8481, Sigma-Aldrich), Suitable for: ELISA (i), IHC, WB; Reacts with: bovine, human, canine, mouse, hamster, chicken

Eukaryotic cell lines

Policy information about cell lines

Cell line source(s)	A murine lymphatic endothelial cell line (mLEC) established from benign lymphangiomas induced by Freund's adjuvant was used, which has been described in the following publication: Sironi, M., et al. Generation and characterization of a mouse lymphatic endothelial cell line. Cell Tissue Res 325, 91-100 (2006). We obtained the mLEC cell line from Dr. Wen Sun (Jiangsu Key Laboratory of Oral Diseases, Nanjing Medical University, Nanjing, PR China).
Authentication	The mLEC cell line was verified in the following publication: Sironi, M., et al. Generation and characterization of a mouse lymphatic endothelial cell line. Cell Tissue Res 325, 91-100 (2006). The mLEC cell line was authenticated by Western blot, enzyme-linked immunosorbent analysis (ELISA), fluorescence-activated cell sorting (FACS) and polymerase chain reaction (PCR).
Mycoplasma contamination	The mLEC cell line had been tested and confirmed negative for mycoplasma contamination.
Commonly misidentified lines (See ICLAC register)	No commonly misidentified cell line was used in this study.

Animals and other organisms

Policy information about studies involving animals; ARRIVE guidelines recommended for reporting animal research

Laboratory animals	FGFR3flox/flox (C3H/HeJ), BMPR1aflox/flox (C3H/HeJ), Col2a1-CreERT2 (C3H/HeJ), Prox1-CreERT2 (C57BL/6j), Prx1-CreERT2 (C57BL/6j), Rosa26tdTomato (C57BL/6j) (Ai14, 007914, Jackson Laboratories, Bar Harbor, ME, USA) and Rosa26mTmG (C57BL/6j) (007676, Jackson Laboratories, Bar Harbor, ME, USA) mice were all previously reported. Prx1-CreERT2, Prox1-CreERT2, Rosa26tdTomato and Rosa26mTmG mice were backcrossed with C3H/HeJ mice for ten generations. 10-week-old male mice were used as described in the manuscript. All mice were bred and maintained under SPF conditions with 12h dark/light cycle, regular chow diet, 24°C temperature and 60% humidity.
Wild animals	No wild animal was involved in this study.
Field-collected samples	No field-collected sample was involved in this study.
Ethics oversight	All animals were maintained and handled with the approval of the Laboratory Animal Welfare and Ethics Committee of Daping Hospital (Chongqing, China).

Note that full information on the approval of the study protocol must also be provided in the manuscript.

Human research participants

Policy information about studies involving human research participants

Population characteristics	HO specimens were collected from male patients who had previously sustained elbow fractures that were treated with

	internal fixation and returned for surgical treatment of acquired HO. All subjects with elbow fractures were previously healthy, nonsmoking males aged between 25 and 45. All patients had no HO treatments including NSAIDs, local irradiation or surgery during the course of our study.
Recruitment	From January 2017 to October 2019, acquired HO specimens were obtained from patients undergoing surgeries in the Department of Trauma Surgery of Daping Hospital. HO specimens were collected from male patients who had previously sustained elbow fractures that were treated with internal fixation and returned for surgical treatment of acquired HO. Osteogenesis stage (3-6 months after fixation surgery) and maturation stage (12-18 months after fixation surgery) were defined by the period since their fixation surgery according to the reference (PMID: 29416028). Only male patients were recruited for a consistent research with animal study.
Ethics oversight	The study was approved by the Ethical and Protocol Review Committee of Daping Hospital (Chongqing, China). All experiments were performed according to approved guidelines. Informed consent had been obtained from all research participants in our study before their surgeries.

Note that full information on the approval of the study protocol must also be provided in the manuscript.
